# The Depsipeptide Romidepsin Reverses HIV-1 Latency *In Vivo*

**DOI:** 10.1371/journal.ppat.1005142

**Published:** 2015-09-17

**Authors:** Ole S. Søgaard, Mette E. Graversen, Steffen Leth, Rikke Olesen, Christel R. Brinkmann, Sara K. Nissen, Anne Sofie Kjaer, Mariane H. Schleimann, Paul W. Denton, William J. Hey-Cunningham, Kersten K. Koelsch, Giuseppe Pantaleo, Kim Krogsgaard, Maja Sommerfelt, Remi Fromentin, Nicolas Chomont, Thomas A. Rasmussen, Lars Østergaard, Martin Tolstrup

**Affiliations:** 1 Department of Infectious Diseases, Aarhus University Hospital, Aarhus, Denmark; 2 Institute of Clinical Medicine, Aarhus University, Aarhus, Denmark; 3 Aarhus Institute for Advanced Studies, Aarhus University, Denmark; 4 Kirby Institute, University of New South Wales Medicine, University of New South Wales Australia, Sydney, Australia; 5 Division of Immunology and Allergy, Lausanne University Hospital, Lausanne, Switzerland; 6 Bionor Pharma ASA, Oslo, Norway; 7 Centre de Recherche du CHUM, Montreal, Quebec, Canada; 8 Department of Microbiology, Infectiology, and Immunology, Université de Montréal, Faculty of Medicine, Montreal, Quebec, Canada; John Hopkins University, UNITED STATES

## Abstract

Pharmacologically-induced activation of replication competent proviruses from latency in the presence of antiretroviral treatment (ART) has been proposed as a step towards curing HIV-1 infection. However, until now, approaches to reverse HIV-1 latency in humans have yielded mixed results. Here, we report a proof-of-concept phase Ib/IIa trial where 6 aviremic HIV-1 infected adults received intravenous 5 mg/m^2^ romidepsin (Celgene) once weekly for 3 weeks while maintaining ART. Lymphocyte histone H3 acetylation, a cellular measure of the pharmacodynamic response to romidepsin, increased rapidly (maximum fold range: 3.7–7.7 relative to baseline) within the first hours following each romidepsin administration. Concurrently, HIV-1 transcription quantified as copies of cell-associated un-spliced HIV-1 RNA increased significantly from baseline during treatment (range of fold-increase: 2.4–5.0; p = 0.03). Plasma HIV-1 RNA increased from <20 copies/mL at baseline to readily quantifiable levels at multiple post-infusion time-points in 5 of 6 patients (range 46–103 copies/mL following the second infusion, p = 0.04). Importantly, romidepsin did not decrease the number of HIV-specific T cells or inhibit T cell cytokine production. Adverse events (all grade 1–2) were consistent with the known side effects of romidepsin. In conclusion, romidepsin safely induced HIV-1 transcription resulting in plasma HIV-1 RNA that was readily detected with standard commercial assays demonstrating that significant reversal of HIV-1 latency *in vivo* is possible without blunting T cell-mediated immune responses. These finding have major implications for future trials aiming to eradicate the HIV-1 reservoir.

**Trial Registration:**

clinicaltrials.gov NTC02092116

## Author Summary

One proposed way of curing HIV is to activate virus transcription and kill latently infected cells while the presence of antiretroviral therapy prevents spreading the infection. Induction of global T cell activation by mitogenic or other potent activators effectively reverses HIV-1 from latency *ex vivo*, but such compounds are generally too toxic for clinical use. Therefore, investigating the capacity of small molecule latency reversing agents to induce production of virus without causing global T cell activation has been a top research priority for scientists in recent years. In the present clinical trial, we demonstrate that significant viral reactivation can be safely induced using the depsipeptide romidepsin (HDAC inhibitor) in long-term suppressed HIV-1 individuals on antiretroviral therapy. Following each romidepsin infusion, we observed clear increases in lymphocyte H3 acetylation, HIV-1 transcription, and plasma HIV-1 RNA. Importantly, this reversal of HIV-1 latency could be measured using standard clinical assays for detection of plasma HIV-1 RNA. Furthermore, romidepsin did not alter the proportion of HIV-specific T cells or inhibit T cell cytokine production which is critically important for future trials combining HDAC inhibitors with interventions (e.g. therapeutic HIV-1 vaccination) designed to enhance killing of latently infected cells.

## Introduction

Antiretroviral therapy (ART) effectively suppresses viral replication and partially restores immune functions in human immunodeficiency virus type-1 (HIV-1) infected individuals [1]. However, HIV-1 integrates into the host DNA, thus establishing the basis for latent infection. As ART cannot eliminate transcriptionally inactive or latent virus, adjunctive interventions that efficiently activate latent virus are needed to achieve the ultimate goal of a cure for HIV-1 infection.

HIV-1 preferentially integrates into the host genome of activated CD4^+^ cells [2, 3]. Upon integration, activation of the host cell transcription machinery leads to the production of new virions. Cells that are actively producing virus typically die rapidly (half-life ~24 hrs) due to virus-induced cytopathic effects and/or immune-mediated killing [4]. However, a minority of memory CD4^+^ T cells carrying replication-competent provirus persists in a resting state during viral suppression by ART [5, 6]. Such transcriptionally silent infected cells are invisible to the host immune system but retain the capacity to reinitiate production of infectious viral particles upon activation. This latent reservoir, likely established within days of infection [7], persists throughout life due to long half-life as well as proliferation of the latently infected memory CD4^+^ T cells [8, 9], and thus represents the primary barrier to an HIV-1 cure [10].

One proposed way of curing HIV is to activate virus transcription and kill latently infected cells in the presence of ART to prevent spreading the infection [11]. Induction of global T cell activation by mitogenic or other potent activators (e.g. PHA, PMA, prostratin) effectively reverses HIV-1 from latency *ex vivo* [12, 13], but such compounds are generally too toxic for clinical use [14]. Therefore, investigating the capacity of small molecule latency reversing agents (LRA) to induce production of virus without causing global T cell activation has been a top research priority for researchers in recent years [15–17]. The ability to induce HIV-1 viremia or at least cell surface expression of viral proteins and presentation of viral antigens is a fundamental requirement for enabling immune mediated killing of latently infected cells and, thus, defines the key goal of LRAs in eradication strategies.

A central mechanism for maintaining HIV-1 latency is the activity of histone deacetylases (HDAC) that represses proviral transcription by promoting histone deacetylation [3, 18]. Several studies have shown that HDAC inhibitors (HDACi) can disrupt HIV-1 latency in vitro [19–21]. There is, however, great variability in the potency among HDACi and very limited data exist on clinical efficacy of various HDACi in reversing HIV-1 latency [15]. Vorinostat, the first potent HDACi to be investigated in a HIV-1 clinical trial [22], increased HIV-1 transcription in CD4^+^ T cells but did not induce plasma HIV-1 RNA in two multiple-dose studies [23, 24]. In contrast, we recently demonstrated that treatment with panobinostat, a hydroxamic acid pan-HDACi like vorinostat, increased both HIV-1 transcription as well as the proportion of plasma samples positive for HIV-1 RNA in 15 ART suppressed participants [25]. Although no significant reductions in the size of the latent HIV-1 reservoir were observed in any of these studies, they demonstrated that HDACi exhibit desired key qualities of LRA including the ability to induce virus transcription *in vivo*. To investigate further the potential of HDACi as the LRA component in the ‘kick and kill approach’ to purge the HIV-1 reservoir, we initiated a study of romidepsin, the most potent HDACi according to *ex vivo* measures [26], to investigate its clinical safety and potential for reversing HIV-1 latency in individuals on long-term ART. In addition, we investigated the impact of romidepsin on T cell activation and function in light of the findings of a recent in vitro study suggesting that HDACi negatively affect cytotoxic T-lymphocytes thus impairing the elimination of HIV-infected cells by [27].

The trial data reported here are the results of the first part of a two-step clinical trial. The objective of the first part was to verify the safety and effect of romidepsin prior to incorporation into the second part comprising combination therapy with romidepsin and the therapeutic HIV-1 vaccine, vacc-4x (Bionor Pharma) [28].

## Results

### Participants enrolled in the study

**Fig 1:**
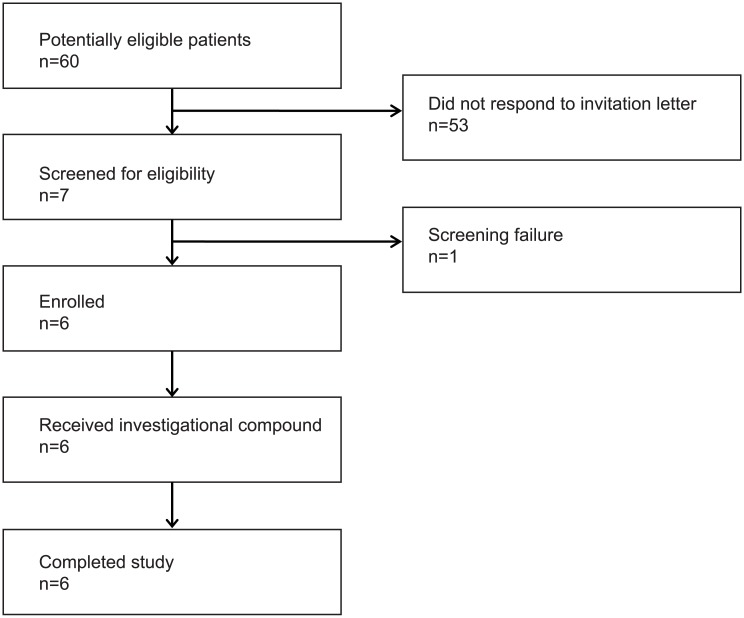
Flow diagram. The flow diagram shows information about the method of recruitment and the number of patients undergoing romidepsin treatment.

Six HIV-1 infected persons (5 male, 1 female) were enrolled in the study (Fig 1). The study participants were all Caucasian and had been on ART for a median of 9.5 years (range 4.2–14.5) with a median CD4^+^ count of 645 cells per μL (range 510–1,000) at inclusion (baseline characteristics shown in Table 1). All six participants received one 4 hour romidepsin infusion (5 mg/m^2^) per week for three consecutive weeks and were followed for up to 70 days after the last infusion.

**Table 1:** Baseline characteristics of the six study participants at enrolment.

Baseline characteristics	n = 6
Male gender, n(%)	5 (83.3)
Caucasian origin, n(%)	6 (100)
Age (years), median (range)	56 (36–60)
Years since HIV diagnosis, median (range)	13.3 (7.0–22.6)
Years from HIV diagnosis to cART initiation, median (range)	1.6 (0.1–18.5)
cART regimen	
TDF, FTC, ATV/r, n(%)	2 (33.3)
TDF, FTC, DRV/r	1 (16.7)
TDF, FTC, RPV	1 (16.7)
TDF, FTC, EFV	1 (16.7)
TDF, FTC, EVG/c	1 (16.7)
Years on cART, median (range)	10.1 (4.1–14.5)
Years with HIV RNA <50 copies per mL, median (range)^A^	9.1 (3.6–12.7)
Nadir CD4+ cell count per μL, median (range)	250 (40–340)
Baseline CD4+ cell count per μL, median (range)	645 (510–1000)

^A^One patient had one blip, 2.3 years prior to inclusion (81 copies/mL), another patient had two blips, latest blip (64 copies/mL) 9.3 years prior to inclusion.

### Romidepsin was safe

The per-protocol defined dose of romidepsin (5 mg/m^2^) had not been investigated previously in HIV patients and corresponded to ~36% of the recommended dosing in cancer treatment (14 mg/m^2^). We observed no severe adverse events (SAEs) or suspected unexpected serious adverse reactions (SUSAR). Forty-one adverse events (AE) were registered during follow-up of which 35 AEs were considered related to romidepsin (Table 2). All drug-related AEs were mild (grade 1, n = 35) and resolved spontaneously within a few days. The number of AEs reported by each study participant during follow-up ranged from 1 to 13. The most common romidepsin-related AEs were abdominal symptoms (e.g. nausea [n = 11], borborygmia [n = 4], abdominal pain [n = 2]) and fatigue (n = 5). Modest changes in white blood cell counts (WBC) and T cell counts were observed during the study (S2 Fig) with the lowest levels generally observed after the second romidepsin infusion, but no further decline following the third infusion. Reassuringly, neutrophil counts below 1000 cells/μL, CD4^+^ cell counts below 350 cells/μL, or platelet counts below 100,000 cells/μL were not observed.

**Table 2:** Self-reported adverse events and their severity during romidepsin treatment.

Type of adverse event (AE)	Grade 1	Grade 2	Any grade	No of patients
*Related to romidepsin*				
Nausea	11	0	11	5
Fatigue	5	0	5	3
Borborygmia	4	0	4	4
Fever/chills	2	0	2	2
Headache	2	0	2	2
Palpitation	2	0	2	1
Heat sensation	2	0	2	1
Reduced appetite	1	0	1	1
Gastrointestinal pain	1	0	1	1
Gastroesophageal reflux	1	0	1	1
Constipation	1	0	1	1
Feeling unwell	1	0	1	1
Intermittent change in sense of taste	1	0	1	1
Intermittent change in sense of smell	1	0	1	1
Any related AE	35	0	35	6
*Not related to romidepsin*				
Dizziness	1	0	1	1
Fracture (accident)	0	1	1	1
Gastrointestinal pain	1	0	1	1
Diarrhea	1	0	1	1
Fever	0	1	1	1
Fatigue	0	1	1	1
Any non-related AE	3	3	6	3

### Cyclic spikes in viral transcription and plasma HIV-1 RNA during romidepsin treatment

**Fig 2:**
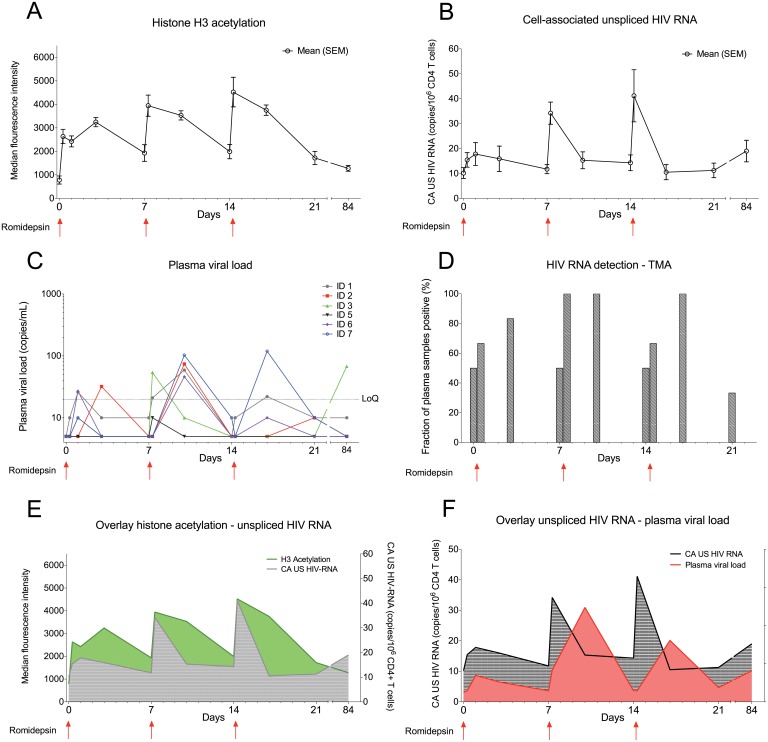
Romidepsin induced HIV-1 transcription and presence of extracellular viral RNA. Panel (**A**) shows mean (SEM) levels of H3 acetylation measured by flow cytometry in lymphocytes. (**B**) shows mean (SEM) change from baseline in the level of CA US HIV-1 RNA. The first sample time point after each of the 3 romidepsin doses is approximately ½ hour after end of the 4 hour infusion. (**C**) shows individual levels of plasma HIV-1 RNA, determined using the Roche Cobas Taqman assay, while panel (**D**) shows mean plasma HIV-1 RNA data for all 6 participants determined using a Transcription-Mediated Amplication assay. **(E)** is an overlay of H3 acetylation and CA US HIV-1 RNA data presented in Panels A and B. (**F**) is an overlay of CA US HIV-1-RNA (**B**) and plasma HIV-1 RNA (**E**). SEM, standard error of mean; CA US HIV-1-RNA: cell-associated unspliced HIV-1 RNA; LoQ, limit of quantitation; TMA, Transcription-Mediated Amplication. Statistical comparisons were performed using Wilcoxon matched-pairs signed-ranks test, Asterisk indicate p<0.05.

The level of histone acetylation is a biomarker of the pharmacodynamic effect of an HDACi on cells [25]. Using flow cytometry, we observed cyclic increases in lymphocyte histone H3 acetylation following each romidepsin infusion confirming the anticipated biological effect of the drug (Fig 2A). The peak level of lymphocyte histone H3 acetylation tended to be higher from the first to third infusion (p = 0.06). Next, as an intracellular measure of HIV-1 transcription in latently infected cells we quantified changes in cell-associated un-spliced HIV-1 RNA (CA US HIV-1 RNA) [29]. In all 6 patients, CA US HIV-1 RNA levels increased significantly from baseline to multiple on-treatment time points (p = 0.03, Fig 2B and S1 Fig). CA US HIV-1 RNA peaked immediately (~½ hr) after the completion of each romidepsin infusion but the increases in HIV-1 transcription were most pronounced after the second and third infusion (maximum fold-increase from baseline ranged from 2.8 to 5.0). We found no association between baseline CA US HIV-1 RNA and the relative increase in HIV-1 transcription (S1 Fig). To determine if the observed increases in HIV-1 transcription also led to the viral particle release into the plasma, we quantified plasma HIV-1 RNA using a standard clinical assay (Roche COBAS TaqMan HIV-1 Test, v2.0, lower limit of quantification of 20 copies/mL). In 5 of 6 patients, plasma HIV-1 RNA increased from undetectable levels at baseline to quantifiable levels (range 21–119 copies/mL) at least once post-infusion (Fig 2C, p = 0.03 for baseline compared to day 10 after first infusion). While two participants had had plasma HIV-1 RNA “blips” of 81 copies/mL and 64 copies/mL 2.3 and 9.3 years prior to inclusion, respectively, the median duration of viral suppression below 50 copies/mL prior to study inclusion was 9.1 years (Table 1). Despite comparable levels of HIV-1 transcription, quantifiable plasma HIV-1 RNA was detected in only 2 of 6 subjects after the third infusion compared to 5 of 6 after the second infusion (p = 0.24). These increases in plasma HIV-1 RNA were subsequently confirmed (Fig 2D) with a transcription mediated amplification (TMA)-based methodology (Procleix Ultrio Plus, Genprobe) that is commonly used for screening donor blood for HIV-1 infection [30]. The detection of quantifiable plasma HIV-1 RNA coincided with romidepsin infusions and generally appeared following increases in lymphocyte H3 acetylation as well as HIV-1 transcription (Fig 2E and 2F). In one participant (ID3, Fig 2C) who initially displayed detectable plasma HIV-1 RNA and then became undetectable by 7 days after the third infusion, we observed quantifiable plasma HIV-1 RNA of 42 and 68 copies/mL at day 56 and 84 post first romidepsin dose, respectively. This individual reported being adherent to ART throughout the study and no external cause for the appearance of quantifiable HIV-1 RNA at the late follow-up visits could be identified. Extended follow-up revealed that by day 112 following the third infusion, plasma HIV-1 RNA had returned to undetectable levels in this individual. Altogether, these data demonstrate that romidepsin had a pronounced and consistent effect on HIV-1 transcription leading to quantifiable levels of plasma HIV-1 RNA using a standard clinical assay.

### The size of the HIV-1 reservoir was unchanged by romidepsin

**Fig 3:**
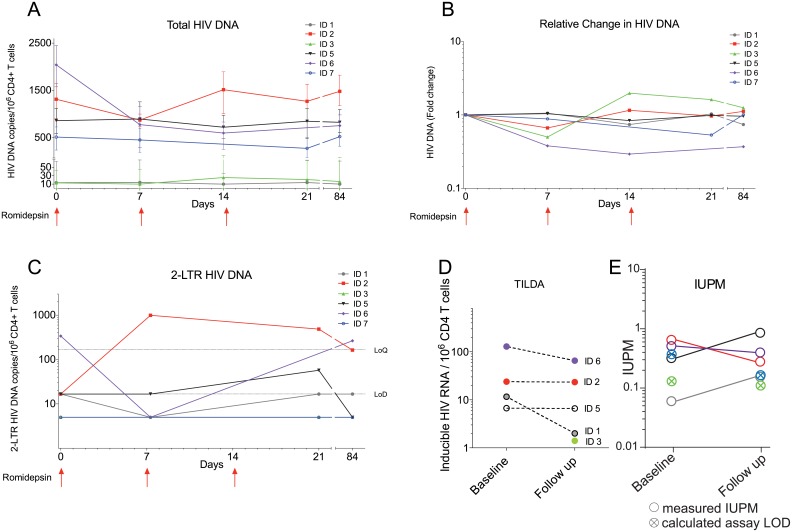
The size of the HIV-1 reservoir was unchanged by romidepsin. Panels (**A)** to (**E)** show measures of the size of the viral reservoir as well as in the inducible HIV-1 reservoir during romidepsin treatment. **(A)** Absolute levels of total HIV-1 DNA per 10^6^ CD4^+^ T cells. (**B**) Fold changes in total HIV-1 DNA per 10^6^ CD4^+^ T cells. **(C)** Absolute levels of 2-LTR HIV-1 DNA per 10^6^ CD4^+^ T cells. **(D)** Frequency of cells with multiply spliced HIV RNA upon maximal cellular activation with PMA/ionomycin as measured using a tat/rev induced limiting dilution assay (TILDA). (**E**) shows results from a quantitative viral outgrowth assay (qVOA) which was used to assess the frequency of resting CD4^+^ cells carrying inducible replication competent proviruses at baseline and day 56. LoQ, limit of quantitation; Lod, limit of detection; IUBM, infectious units per million.

To determine if reversal of HIV-1 latency by romidepsin impacted the viral reservoir, we first used qPCR-based assays to measure total HIV-1 DNA and 2-LTR episomes in CD4^+^ T cells isolated from the 6 participants. Although one participant had a 67% decline in total HIV-1 DNA from baseline to last follow-up, we observed no overall change in total HIV-1 DNA levels indicating that the frequency of CD4^+^ T cells harboring total HIV DNA remained stable following romidepsin administration (Fig 3A and 3B). On a cohort level, the amount of 2-LTR HIV-1 DNA also did not change during the study (Fig 3C). Of note, a large increase in 2-LTR HIV-1 DNA was observed in the individual receiving integrase-inhibitor based combination ART. Next, we used the novel Tat/rev Induced Limiting Dilution Assay (TILDA) to measure the frequency of cells with multiply spliced HIV RNA upon maximal cellular activation with PMA/ionomycin before and after romidepsin treatment (Fig 3D). Four of 4 (100%) participants with available samples, were TILDA positive before romidepsin treatment, and 5 of 6 (83.3%) patients were TILDA positive 6 weeks after the third romidepsin infusion. In the four participants with available samples before romidepsin, 2 of 4 participants had stable numbers of positive events in the TILDA assay from pre/on romidepsin levels to 6 weeks after the last infusion (1–2% decrease) whereas 2 of 4 participants had moderate 49–83% decreases. Finally, we used a quantitative viral outgrowth assay (qVOA) to assess changes in the frequency of resting CD4^+^ cells carrying inducible replication competent proviruses. We found no significant changes the replication competent reservoir from baseline to 6 weeks after the third romidepsin infusion as measured by qVOA (Fig 3E and S1 Table). Overall, we found no substantial reduction in the frequency of cells harboring total HIV-1 DNA or in the size of the inducible replication-competent HIV-1 reservoir following romidepsin treatment.

### Romidepsin increased T cell activation and lowered PD-1 expression

To assess the effects of romidepsin on differentiation and activation status of T cells, we performed flow cytometry analyses at day one, day 10, and day 56 after the first romidepsin infusion. First, we observed changes in the relative proportions of both CD4^+^ and CD8^+^ T cell memory subsets. The mean frequency of naïve CD4^+^ T cells increased from 47.3% at baseline to 56.9% day one after the first dose (p = 0.03) (Fig 3A). These frequencies had returned to pre-dosing levels at the subsequent time points. Similarly, the frequency of naïve CD8^+^ T cells significantly increased 24 hours after the first dose (baseline 19.3% to 31% on day 1, Fig 3B).

**Fig 4:**
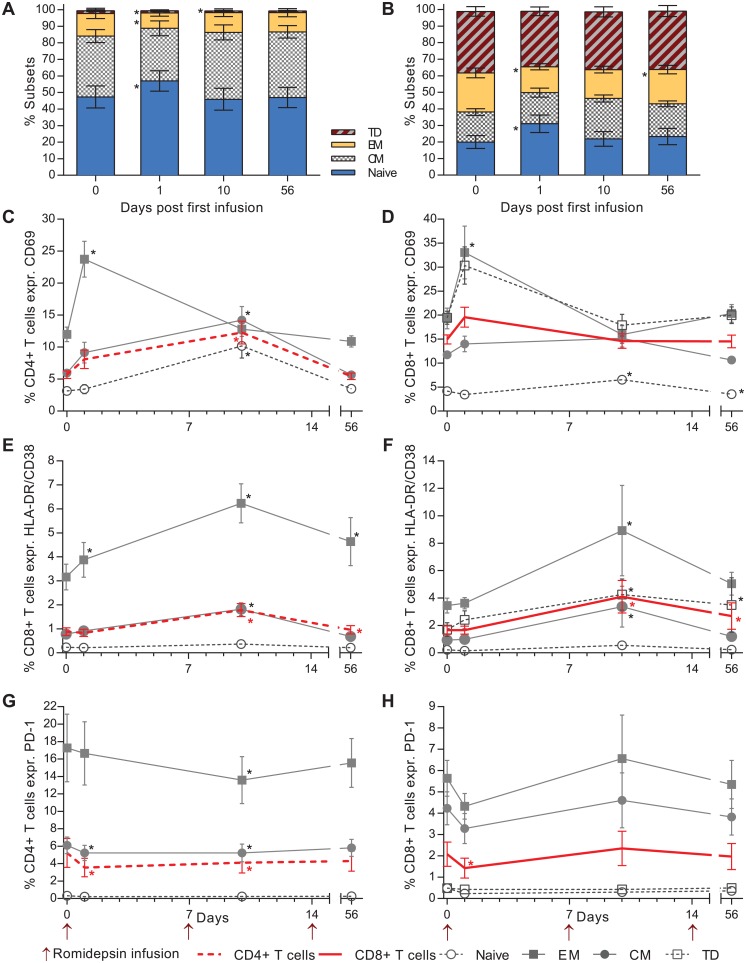
Romidepsin increased T cell activation and reduced the proportion of lymphocytes expressing PD-1. Flow cytometric characterization of CD4^+^ / CD8^+^ T cell subsets and activation status. Relative proportions of both CD4^+^ **(A)** and CD8^+^ **(B)** T cell memory subsets are shown in the first two panels. Next, the proportion of CD4^+^ **(C)** and CD8^+^ **(D)** T cells expressing the early activation marker CD69. **(E)** Percentage of CD4^+^ and **(F)** CD8^+^ T cells co-expressing the late activation markers HLA-DR and CD38. (**G, H)** Show the effect of romidepsin on the proportion of CD4^+^ and CD8^+^ T cells expressing the exhaustion marker PD-1. CM, central memory; EM, effector memory TD; terminally differentiated. Statistical comparisons were performed using Wilcoxon matched-pairs signed-ranks test, Asterisk indicate p<0.05.

T cell activation was evaluated by measuring the frequency of cells expressing CD69 or co-expressing HLA-DR and CD38 (gating strategy depicted in S3 Fig). The percentage of both CD4^+^ and CD8^+^ T cells expressing CD69 increased substantially at 24 hours after the first dose (Fig 4C and 4D).

The largest increase in percentage of cells expressing CD69 was observed in the effector memory (EM) CD4^+^ T cells (mean increase from 11.9% to 23.7% CD69^+^, Fig 4C) and terminally differentiated (TD) CD8^+^ T cells (Fig 4D). Similarly, the proportion of CD4^+^ and CD8^+^ cells co-expressing HLA-DR/CD38 increased significantly over baseline at three days following the second infusion (Fig 4E and 4F). As observed for CD69, the greatest increase in HLA-DR/CD38 co-expressing CD4^+^ and CD8^+^ T cells was observed in the effector memory compartment. Further, we evaluated the effect of romidepsin on frequency of CD4^+^ and CD8^+^ T cells expressing the exhaustion marker PD-1. Day one after the first infusion there was a significant decrease in the frequency of both CD4^+^ and CD8^+^ T cells expressing PD-1 and for the CD4^+^ T cells there was also a reduction at day 10 after the first infusion (Fig 4G and 4H). These early changes in the frequency of cells expressing PD-1 which were found across all memory subsets had returned to pre-dosing baseline levels at day 56 after the first infusion.

### T cell responses to antigen stimulation were intact during romidepsin treatment

**Fig 5:**
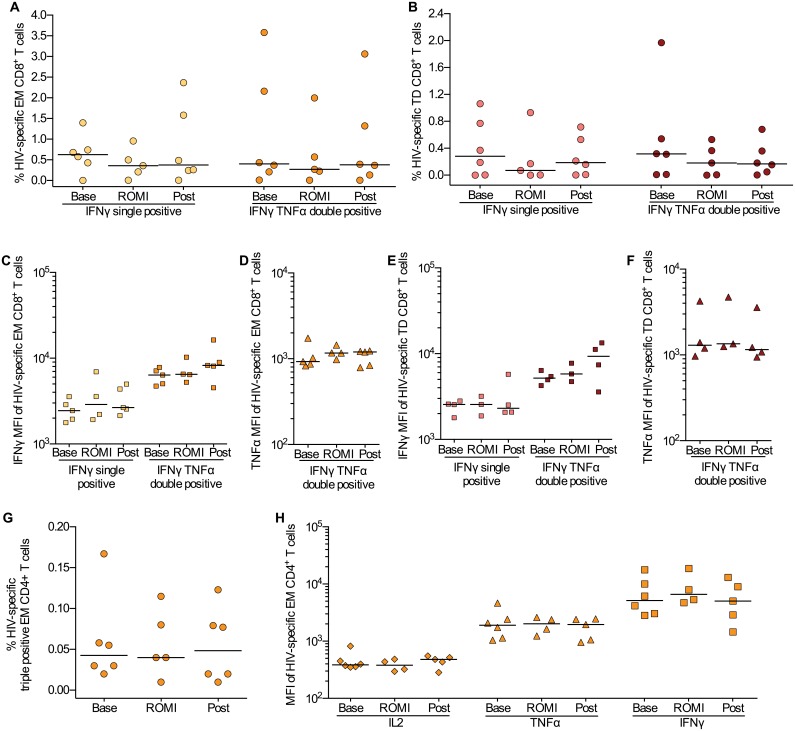
HIV-specific T cell responses were preserved during romidepsin treatment. Flow cytometric characterization of HIV-gag-specific CD8^+^ and CD4^+^ T cells within the memory subsets at baseline (Base, *n* = 6), on treatment (ROMI, *n* = 5) and at follow-up (Post, *n* = 6). Proportion of EM (**A**) and TD (**B**) CD8^+^ T cells producing only IFNγ or both IFNγ and TNFα. Horizontal bars show median values. **(C, D)** Median fluorescence intensity (MFI) for IFNγ and TNFα for HIV-specific EM CD8^+^ T cells and **(E, F)** TD CD8^+^ T cells identified in Panels A-B. **(G)**, Proportion of polyfunctional memory EM CD4^+^ T cells producing IFNγ, TNFα and IL-2. **(H)** Expression levels (MFI) for each cytokine (i.e. IL-2, TNFα and IFNγ) examined on the polyfunctional HIV-specific EM CD4^+^ T cells identified in panel **(G)** TD, terminally differentiated; EM, effector memory. Statistical comparisons were performed using Wilcoxon matched-pairs signed-ranks test, Asterisk indicate p<0.05.

In vitro data suggests that HDACi, and romidepsin in particular, may induce or intensify T cell dysfunction compromising the clearance of virus-producing cells [27]. To investigate whether this finding could be recapitulated during clinical administration, we first evaluated the impact of romidepsin on the function of HIV-1-specific CD4^+^ and CD8^+^ T cells. Following *ex vivo* stimulation of PBMCs with a library of 150 overlapping HIV-gag peptides, we performed intracellular cytokine staining (ICS) for IFN-γ, TNF-α and IL-2 (S4 Fig). The HIV-1-specific CD8^+^ T cells primarily exhibited an EM or TD phenotype and produced solely IFN-γ or both IFN-γ and TNF-α. The majority of HIV-1-specific CD4^+^ T was EM cells and co-produced all three cytokines analyzed. The ICS analyses showed no negative impact on the CD4^+^ or CD8^+^ T cell capacity to produce IFNγ, TNFα, or IL-2 following romidepsin administration, both with regard to the frequency (%) of HIV-gag specific EM CD4^+^ and EM and TD CD8^+^ T cells present and with regard to the levels (MFI) of cytokines produced by the individual cells (Fig 5A–5H). We also found no change in non-HIV-1 specific CD4^+^ and CD8^+^ T cell responses to staphylococcal enterotoxin b (SEB) during romidepsin treatment (S5 Fig). Similar to the flow cytometry-based measures, ELIspot analyses with overlapping HIV p24 gag peptides showed no change in functional responses following romidepsin treatment (S6 Fig). Collectively, these data indicate that romidepsin treatment had no detrimental effect on HIV-1-specific and general T cell immunity in this study.

## Discussion

Herein, we demonstrated that significant viral reactivation can be safely induced using the HDACi romidepsin in long-term suppressed HIV-1 individuals on ART. The cyclic increases in lymphocyte H3 acetylation, HIV-1 transcription, and plasma HIV-1 RNA following infusion of romidepsin link the events leading from romidepsin infusion, via epigenetic modification and induction of HIV-1 transcription, to increases in plasma HIV-1 RNA. Importantly, this reversal of HIV-1 latency was measured using standard clinical assays for detection of HIV-1 RNA in plasma. Furthermore and very important from a safety perspective, romidepsin did not alter the proportion of HIV-1-specific T cells or inhibit T cell cytokine production. However, despite the increases in viral production and preserved T cell functions, no substantial changes in the size of the HIV-1 reservoir were observed.

Previous clinical trials provide support for the use of HDACi to safely disrupt HIV-1 latency *in vivo*; however, the magnitude of viral induction in the present study was greater than anything previously reported for any LRA tested in humans. In the pioneering study by Archin et al. [22], a single 400 mg oral dose of the HDACi vorinostat produced a median 4.6 fold increase in HIV-1 transcription in resting memory CD4^+^ T cells. Despite the clear increase in HIV-1 transcription, no increase in plasma HIV-1 RNA could be detected using the ultrasensitive single copy assay [22, 31]. Subsequent multi-dose studies have confirmed an effect of vorinostat on HIV-1 transcription but no consistent changes in plasma HIV-1 RNA were detected in these studies [23, 24] suggesting that post-transcriptional blocks may mitigate the effect of vorinostat as an LRA[32]. Further, a recent publication questioned whether any of the clinically available LRAs would be potent enough to reverse latency when administered individually [13]. The present study demonstrated potent *in vivo* latency reversal with a single drug resulting in increased plasma HIV-1 RNA that was readily quantified with standard commercial assays. However, the exact proportion of infected cells reversed from latency out of the total pool of inducible latently infected cells remains unknown.

Another key observation in the present study was the unambiguous increase in HIV-1 transcription and release of viral particles following the second romidepsin infusion as compared to following the first romidepsin infusion. Clearly, the hyper-acetylated state induced by the first romidepsin infusion (Fig 2B) did not reach baseline level before the second romidepsin infusion. Therefore, the effect of the second romidepsin infusion may add to the sustained effects of the first infusion, which might explain the more pronounced effect on HIV-1 transcription and viral particle release. It is likely that on a single cell basis HIV-1 transcription needs to cross a certain threshold to overcome transcriptional blocks before it gives rise to viral protein translation and virion production [3]. Such an outcome would be consistent with the increase in both CA US and plasma HIV-1 RNA following the second infusion.

Interestingly, lymphocyte H3 histone acetylation and CA US HIV-1 RNA levels further increased following the third infusion, compared to after the second infusion, whereas plasma HIV-1 RNA did not. Up-regulation of intracellular factors that block viral translation, unknown effects of romidepsin or the induction of intracellular antiviral immune responses towards HIV-1 could also account for this observation but larger studies are needed to shed light on whether this potential difference in response between the second and third infusion is biologically relevant. In addition, indirect effects of romidepsin on CD4+ T cells such as more generalized activation (as measured by CD69 expression) may also play a role in the observed increases in HIV transcription.

HDACs are epigenetic regulators with the capacity to alter the expression of genes involved in a broad range of immune cell functions, thus affecting their interaction with and responsiveness to pathogens [33]. In vitro studies have suggested that HDACi in general and romidepsin in particular inhibit HIV-1 specific T cell immunity leading to impaired clearance of virus-producing cells [27]. In this study, we examined the participants’ cellular immunity and found no evidence for suppression of HIV-1-specific or non-HIV-1-specific CD4^+^ or CD8^+^ T cell immunity during or after romidepsin treatment. Our clinical observations are in line with other findings from anti-tumor experiments showing that T and NK cells are resistant to the immunosuppressive functions of HDACi [34]. Further, in our study expression of the negative regulator of immune responses, PD-1 on CD4^+^ and CD8^+^ T cells, decreased significantly while T cell activation increased suggesting that romidepsin did not impair T cell reactivity. Collectively, our findings strongly suggest that romidepsin does not negatively affect T cell functions *in vivo* which is critically important for future trials combining HDACi with interventions (e.g. therapeutic HIV-1 vaccination) designed to enhance CTL-mediated killing of latently infected cells. One such trial combining romidepsin and therapeutic HIV-1 vaccination using vacc-4x is currently under way as part B of the present study in our clinic (see http://clinicaltrials.gov NTC02092116).

In the current study, we found no significant effect of romidepsin treatment on the size of the HIV-1 reservoir when measuring total HIV-1 DNA, the frequency of cells with multiply spliced HIV RNA upon maximal cellular activation with PMA/ionomycin according or by qVOA. Whether this persistence of a functional reservoir is due to incomplete latency reversal and/or insufficient clearance of reactivated cells (e.g. due to CTL-resistant viruses as recently suggested [35]) remains to be investigated.

In general, the safety and tolerability of HDACi in latency reversal trials has been good. Nevertheless, due to the broad epigenetic effects of HDACi including the potential to modulate signaling pathways and the expression of numerous proteins, careful monitoring of adverse events is essential [23–25]. In accordance with experiences in oncology patients [36], the most frequently reported adverse effects of romidepsin in HIV-infected persons were abdominal symptoms and fatigue. While these symptoms were common in this study, they were generally mild and did not lead to dose de-escalations or study withdrawals according to the pre-specified protocol criteria. In addition to potential AE that lead to patient discomfort, animal and in vitro studies indicate that HDACi could potentially interfere with the function of other immune cells such as plasmacytoid dendritic cells, macrophages, and neutrophils, leading to increased risk of infection [27, 37]. Reassuringly, in our trial and in previous trials using HDACi treatment to reverse HIV-1 latency, an increased risk of infections among study participants has not been observed [23–25]. Further, we did not observe clinically significant or persistent decreases in neutrophil, monocyte, CD4^+^, or CD8^+^ counts. Collectively, our data suggest that the utilized romidepsin dosing schedule has an acceptable safety profile in HIV-1-infected persons and does not result in persistent changes in blood biochemistry levels.

While our findings can be used as a basis for investigating romidepsin in future HIV eradication trials, the limitations of this study should also be acknowledged. First, the small sample size limited the strength of our statistical analyses. Despite this limitation, the cyclic appearance and near-identical inter-individual patterns of HIV-1 transcription and plasma HIV-1 RNA levels, clearly show that pronounced viral reactivation does occur during romidepsin treatment in ART treated HIV-1 subjects. Second, our study only included Caucasians and care should be taken when generalizing the results to other populations as responses to romidepsin may differ among populations [38].

In summary, in this study we demonstrated significant reversal of HIV-1 latency following romidepsin infusions. Romidepsin-induced increases in HIV-1 transcription were followed by increases in readily measurable plasma HIV-1 RNA. Plasma HIV-1 RNA peaked at levels readily quantifiable with certified clinical assays thus establishing a new benchmark for future trials investigating the *in vivo* potency of LRAs to be used in HIV-1 eradication efforts.

## Methods

### Trial design and study participants

We conducted this single-arm, single-site, phase Ib/IIa clinical trial at Aarhus University Hospital, Denmark as a first step of a two-step clinical study between March 2014 and July 2014. This study enrolled HIV-1 infected adults on ART with virological suppression for at least one year (<50 copies/mL, minimum 2 measurements per year) and CD4^+^ T cell counts above 500/μL at inclusion. Major exclusion criteria included: hepatitis B or C co-infection; clinically significant cardiac disease including QTc-prolongation; any significant acute medical illness in the 8 weeks prior to inclusion; unacceptable values of the hematologic and clinical chemistry; history of malignancy; or diabetes. Full details regarding inclusion/exclusion criteria can be found at http://clinicaltrials.gov.

### Patient procedures

Approximately one hour prior to romidepsin treatment, a blood draw was performed. Hematologic and clinical chemistry was checked pre-infusion and additional material stored for endpoint analyses. Thirty minutes prior to infusion patients received 8 mg ondensatron as prophylactic antiemetic treatment. Patients received romidepsin (5 mg/m^2^) administered intravenously over a 4 hour period once weekly for three consecutive weeks while maintaining ART. This dose regimen corresponds to the 28-day cyclic regimen used to treat T cell lymphomas but at ~36% of the recommended oncology dose (14 mg/m^2^). The dose of 5 mg/m^2^ was chosen based upon extensive pre-clinical *ex vivo* testing of the ability of romidepsin to induce HIV-1 production in latently infected resting CD4^+^ T cells isolated from ART suppressed HIV patients [26]. Of note, romidepsin is metabolized through CYP3A4 and inhibitors or inducers of this enzyme could affect romidepsin exposure. In a pharmacokinetic drug interaction trial the strong CYP3A4 inhibitor ketoconazole increased the overall romidepsin exposure by approximately 25% and 10% for area under the curve (AUC)0-∞ and peak exposure (Cmax), respectively, compared to romidepsin alone [39]. Thus, co-administration of ketoconazole slightly decreased the romidepsin clearance and volume of distribution, but did not have a statistically significant effect on Cmax. While ketoconazole is classified by FDA as a strong in vivo inhibitor of CYP3A4, protease inhibitors such as darunavir/ritonavir and atazanavir are classified as moderate inhibitors of CYP3A4 whereas efavirenz is classified as a moderate inducer of CYP3A4 [40]. Based on a thorough evaluation of the romidepsin investigator’s brochure, we estimated that the risk of clinically significant drug-drug interaction was extremely low and that a potential interaction between antiretroviral drugs and romidepsin was very unlikely to cause >25% increase/decrease in romidepsin exposure.

Including the baseline, on therapy and follow up visits, there were a total of 13 study visits. Blood was drawn at each visit including 1 hour prior to and 1/2 hour after receipt of each dose, 24 and 72 hours after the first dose, 72 hours after the second and third dose, as well as 1, 6, and 10 weeks after completion of romidepsin. At each follow-up visit, self-reported adherence to ART was recorded.

Safety assessments were actively performed during on all study visits. This included recording all patient-reported adverse events (AEs) and serious adverse events (SAEs). For each AE/SAE the relationship to romidepsin was evaluated and the severity graded according to the Common Terminology Criteria for Adverse Events (CTCAE) version 4.0.

### Endpoints

The pre-specified primary endpoint was safety and tolerability of romidepsin at a reduced dosing of 5 mg/m^2^ in HIV-1 infected patients. Secondary endpoints included change from baseline in HIV-1 transcription according to CA US HIV-1 RNA measures in unfractionated CD4^+^ T cells, change from baseline in plasma HIV-1 RNA, and change from baseline in total HIV-1 DNA per 10^6^ CD4^+^ T cells. Changes from baseline in T cell activation markers, T cell subset distribution, and PD-1 expression as well as changes from baseline in HIV-1-specific and non-HIV-1-specific T cell immunity were exploratory immunological endpoints. Change from baseline in the frequency of cells with multiply spliced HIV RNA upon maximal cellular activation with PMA/ionomycin using a novel assay Tat/rev Induced Limiting Dilution Assay (TILDA) and 2-long terminal repeat (LTR) circles per 10^6^ CD4^+^ T cells were exploratory virological endpoints.

### Cell-associated un-spliced HIV-1 RNA

For quantification of CA US HIV-1 RNA, CD4^+^ T-cells were isolated from peripheral blood mononuclear cells (PBMC) using a CD4^+^ T-cell isolation kit and magnetic-activated cell sorting (MACS) columns (Miltenyi Biotec, Teterow, Germany; purity >95%). Isolated CD4^+^ cells were lysed and lysates were stored at -80°C until RNA and DNA was extracted (Allprep isolation kit, Qiagen). Template preparation and denaturation was performed in 13.5μL reaction volume containing a mixture of 11.5μL patient extracted RNA, 1μL of 10mM deoxynucleoside triphosphates mix, 1.5 μg of random primers and 0.25 μg Oligo(dT)12-18 primer (LifeTechnologies, Denmark) at 65°C for 5 min followed by immediate incubation on ice for 5 min. First-strand cDNA production was performed by adding a mixture of 4 μL 5X first-strand buffer (250 mM Tris-HCl (pH 8.3), 375 mM KCl, 15 mM MgCl2), 1μL of 0.1M DTT, 20 U RNaseOUT Recombinant RNase Inhibitor and 200 units of SuperScriptTM III reverse transcriptase (LifeTechnologies, Denmark). Reverse transcription was performed in the resulting 20μL total reaction volume at 42°C for 45 minutes followed by heat inactivation of the reverse transcriptase at 80°C for 15 minutes.

The ddPCR mixture for the CA US HIV-1 RNA assay consisted of: 11 μL 2x digital PCR supermix (BioRad, DK), 3 μL of primer/probe (primers SL19/20 final concentration1000nM and MGB probe SL30MIDDLE 5’-TACTCACCAGTCGCCGC-3 final concentration 250nM, 5μL nuclease-free water and 3μL patient derived cDNA resulting in 22μL reaction volume. To adjust for the total cellular input in each sample, relative copy numbers were normalized to two human endogenous control genes: TBP PL (VIC) assay ID—Hs00183533_m1 and IPO8 (FAM) assay ID—Hs00427620_m1 (TaqMan gene expression assay, LifeTechnologies, Denmark). All HIV RT samples were assayed in six replicates while the reference genes were assayed in duplicate. The PCR reaction mixture was loaded into the BioRad QX- 100 emulsification device fractionating each sample into ~20,000 nanoliter-sized droplets following the manufacturer’s instructions. PCR cycling conditions were as follows: 95°C for 10 min, followed by 40 cycles of a 30 second denaturation at 95°C followed by a 59°C extension for 60 seconds and a final 10 minutes at 98°C. After cycling droplets were subsequently read automatically by the QX100 droplet reader (BioRad) and the data was analyzed with the QuantaSoftTM analysis software (BioRad). The six HIV replicates generated 80,000–98,000 droplets to be analyzed per time point.

### Plasma HIV-1 RNA

The presence of HIV-1 RNA in EDTA plasma was quantified with the Cobas TaqMan HIV-1 Test, v2.0 (Roche) according the manufacturer’s instruction. This assay has a lower limit of quantification of 20 copies HIV-1 RNA/mL but can provide a qualitative assessment of the presence of HIV-1 RNA below the 20 copy range. The presence of HIV-1 RNA in plasma was also qualitatively assessed using nucleic acid testing using a transcription-mediated amplification (TMA) based detection method as described by the manufacturer (Procleix Ultrio Plus, Novartis) with 50% sensitivity at 3.8 copies/mL and 95% sensitivity at 12 copies/mL [30]. TMA results were considered binary and defined as positive or negative according to assay outcomes.

### Quantifications of cell-associated HIV-1 DNA

For HIV-1 DNA quantifications, CD4^+^ T cells were isolated using a CD4^+^ T Cell Isolation Kit Miltenyi biotec, cat no 130-096-533) on LS columns (Miltenyi biotec, cat no 130-042-401). After CD4^+^ T isolation, cells were resuspended in lysis buffer and digested as previously described [41]. Cell lysates were used directly for HIV-1 DNA quantifications using the QX100 Droplet Digital PCR system (Bio-Rad) to determine the absolute levels of total HIV-1 DNA per 10^6^ CD4^+^ T cells. HIV-1 2-LTR circles where quantified as previously described [42].

### Inducible virus quantification using Tat Rev Inducible Limiting Dilution Assay (TILDA)

CD4^+^ T cells were isolated from PBMCs from study participants by negative magnetic selection (StemCell), and stimulated with phorbol myristate acetate (PMA; 100ng/mL) and ionomycin (1μg/mL) for 12hrs. Dilutions of the stimulated cells (ranging from 18,000 to 1,000 cells, 24 replicates per dilution) were distributed in a 96 well plate and directly subjected to RT-PCR. Multiply spliced HIV RNA was quantified by semi-nested real time PCR with primers in tat and rev as previously described [43] with some minor modifications. The frequency of positive cells was calculated using the maximum likelihood method [44] and this number was then expressed as a frequency of cells with inducible multiply spliced HIV RNA per million CD4^+^ T-cells.

### Viral outgrowth assay

Viral outgrowth assays were performed as previously described with the following modifications [25, 45–47]. Resting CD4^+^ T cells were enriched from 150 million cryopreserved PBMCs by negative depletion via a 2-step protocol [45]. Briefly, the first step was to enrich CD4^+^ T cells from PBMCs using Miltenyi CD4^+^ T Cell Isolation Kit (Cat #: 130-096-533) according to the manufacturer’s protocol. The second step was to further enrich for resting CD4^+^ T cells via depletion of cells expressing CD69, CD25 or HLA-DR (Miltenyi Cat #: CD69 Microbeads Kit II– 130-092-355; CD25 Microbeads II– 130-092-983; HLA-DR Microbeads– 130-046-101). Resting CD4^+^ T cell purity, as determined by flow cytometry, was 98% [mean value with a 95% CI of 97.03 to 98.97%]. All cell incubations at 37°C unless otherwise noted. The culture medium for this assay was RPMI with L-glutamine; 1% streptomycin and penicillin; 10% fetal calf serum; recombinant human IL-2 (100 U/mL) (Gibco #PHC0027); conditioned media from a mix lymphocyte reaction culture as described in [46].

On day 0 resting CD4^+^ T cells were seeded at 20,000 cells/well in round-bottom 96-well plates and stimulated with irradiated allogeneic PBMCs from HIV-negative healthy donors and phytohemagglutinin (1μg/mL) (PHA; Remel #R30852801). After 48 hours, the cells were extensively washed to remove PHA and 10,000 MOLT-4/CCR5^+^ cells were added to each well. On days 5, 7, and 9, 75% of the culture media per well was replenished with fresh media with an additional 10,000 MOLT-4/CCR5^+^ cells added to each well with the fresh media on day 9. On day 12, the cell supernatant from each well was harvested and the number of wells containing replication competent HIV was assessed by incubation of the supernatant with TZM-bl cells via firefly luciferase reporter gene activity [48]. On day 15, wells positive for luciferase activity was determined using the Britelite plus Reporter Gene Assay System, 100 mL (Perkin Elmer #: 6066761). Estimated frequencies of cells with replication-competent HIV before and after romidepsin treatment were calculated using limiting dilution analysis as described in [44].

### Detection of histone acetylation levels

Flow cytometry for histone acetylation levels was based on the method developed by Rigby et al. [49]. Immediately after isolation 1x10^6^ PBMCs were re-suspended in 3 mL ice-cold PBS containing 1% FBS, centrifuged and re-suspended for fixation in 200μL 1% paraformaldehyde. The cells were fixed on ice for 15 min, washed in 4 mL ice-cold PBS, re-suspended in 200μL of PBS, and stored at 4°C. Within one week cells were washed with PBS containing 2% FBS and permeabilized with 200μL of 0.1% Triton X-100 in PBS for 10 min. at room temperature. After washing with PBS/2% FBS, the samples were blocked in 600μL of PBS/10% FBS for 20 min. Samples were stained with polyclonal rabbit anti-acetyl histone H3 (10μg/mL) (Merck Millipore #06–599) or normal rabbit serum (control stain) (LifeTechnologies #10510) for 1 hour and then washed and incubated with donkey anti-rabbit IgG (H+L) Alexa Flour 488 (6μg/mL) (LifeTechnologies #A21206) for 1 hour at room temperature in the dark. Cells were washed, re-suspended in 150μL PBS and analyzed. ~50,000 events were acquired per sample. The median fluorescence intensity (MFI) for each patient at each time point was derived via subtraction of the background MFI (isotype control stain) for each sample from the anti-acetyl histone H3 stain.

### T cell activation, subsets, and PD-1 expression

Frozen PBMC’s were thawed and 5x10^5^ cells were immediately stained with Near-IR live-dead dye (LifeTechnologies, Denmark), blocked and then stained with antibodies to CD4-PE-Cy7 (SK3), CD8^+^-PerCP-Cy5.5 (SK1), CD45RA (HI100), CCR7 (G043H7) CD69-APC (FN50), HLA-DR-PE (G46-6) and CD38-BV605 (HB7) or PD-1 (EH12.1) (all Biolegend except PD-1, CD38, HLA-DR and CD8^+^; BD Bioscience). Only singlet, live cells were included in the data analyses. T cells were gated based upon size and granularity (lymphocyte gate). Within the lymphocyte gate T cells were sub-gated based upon their expression of either CD4 or CD8^+^. Memory subsets within CD4^+^ and CD8^+^ T cells were defined based on CCR7 and CD45RA expression. Activation status was determined based upon CD69 expression or HLA-DR/CD38 co-expression. Gates for activation markers and PD-1 were determined using isotope control antibodies.

### HIV-1- specific and SEB-specific T cell immunity

Cryo-preserved PBMCs were analyzed using intracellular cytokine staining (ICS). PBMCs were thawed and rested for 18 hours at 37°C, 5%CO_2_. Next, PBMCs were washed, counted and resuspended at a concentration of 3.3x10^6^ cells/mL in total volume of 0.6 mL for each condition. PBMCs were stimulated for 6 hours at 37°C, 5%CO_2_ with HIV-1 Gag peptide pool (150 peptides mix, final conc. 2μg/mL per peptide, IPT, PepMix HIV (GAG) Ultra) in the presence of secretion inhibitors (Golgistop at 0.7μL/mL, and Golgiplug at 1μL/mL, BD) and co-stimulatory molecules (αCD28, and αCD49d, at 1μg/mL each, BD). Un-stimulated and positive control samples (staphylococcal enterotoxin b, SEB at 1μg/mL, Sigma-Aldrich) were included for each time point. After the stimulation, cells were stained with Near-IR amino reactive dye for viability (Invitrogen) followed by surface staining (including CD4^+^ (OKT4) and CCR7 (G043H7) from Biolegend and CD8^+^ (RPA-T8) and CD45RA (HI100) from BD) and intracellular cytokine staining (IFNγ (B27), IL-2 (MQ1-17H12), TNFα (Mab11) from Biolegend) using BD Cytofix/Cytoperm protocol. ~800,000 events were collected per sample. Gating strategy for analyzing CD8^+^ and CD4^+^ T cell responses in memory subsets are illustrated in S3 Fig. After defining gates for positive IFNγ, TNFα, and IL-2 expression, we utilized Boolean combination gate analyses to create the full array of possible combinations (7 response patterns for 3 functions). Fluorescence minus one (FMO) controls were performed for surface marker CCR7 and intracellular markers IFNγ, IL-2, TNFα. Based on background cytokine response in un-stimulated control samples a positive HIV-1-specific response for CD8^+^ and CD4^+^ T cells was defined as values greater than 0.05% and 0.015% respectively (after background response in the un-stimulated control was subtracted. For the analyses of MFI values (S4 Fig) only samples with a positive HIV response were included. All samples were analyzed on a BD FACSVerse cytometer and data was analyzed using FlowJo Version 10.0.7. T-cell proliferation assays and ELISPOT (to detect γ-interferon were carried using overlapping 15-mer peptides to HIV p24 synthesized at Schafer-N (Copenhagen, Denmark) and staphylococcal enterotoxin B as positive control (Sigma-Aldrich AG, St. Louis MO, USA) as antigens according to the methods described by Pollard et al. [28]. ELISPOT results were considered valid if the mean of triplicate wells did not exceed 50 spot forming units (sfu)/10^6^ cells and the positive control was >500 sfu/10^6^ cells.

### Statistical analyses

The study was designed to determine the safety profile of a reduced romidepsin dose regimen in HIV-1 patients on ART. Baseline characteristics were tabulated and adverse events graded according to CTCAE criteria. Changes from baseline to specific time points were tested using paired t-test or Wilcoxon signed-rank test. Delta values were assessed using the binomial test (two-sided).

### Ethics statement

The study was approved by the Danish Health and Medical Authorities as well as the Danish Data Protection Agency. Ethics committee approval was obtained in accordance with the principles of the Helsinki Declaration. Each patient provided written informed consent prior to any study procedures. The trial is registered at http://clinicaltrials.gov (NTC02092116)

## Supporting Information

S1 Fig Individual levels of cell-associated unspliced HIV-1 RNA. (EPS)

S2 Fig CD4^+^, CD8^+^, and white blood cell counts during romidepsin study. Wilcoxon matched-pairs signed-ranks test, Asterisk indicate p<0.05.
(TIF)

S3 Fig Gating strategy for activation markers CD69, HLA-DR, CD38, and exhaustion marker PD-1. Shown are representative dot plots for patient 2. Samples were analyzed using the following gating strategy for identifying T cell activation and PD-1 expression: Live gate (SSC versus NEAR IR viability stain) → Singlet gate (FSC-A vs. FSC-H) → Lymphocyte gate (SSC-A vs. FSC-A) → CD8^+^ or CD4^+^ T cells (CD8^+^ vs. CD4^+^) and then CCR7 versus CD45RA to define memory subsets within both the CD8^+^ and CD4^+^ T cell populations; naïve (CD45RA^+^CCR7^+^), central memory (CM) (CD45RA^-^CCR7^+^), effector memory (EM) (CD45RA^-^CCR7^-^) and terminally differentiated (TD) (CD45RA^+^CCR7^-^). Finally, within each of the CD8^+^ and CD4^+^ memory subsets we gated on CD8^+^ and CD4^+^ respectively versus CD69, HLA-DR, CD38 or PD-1. Numbers represent percentage of the shown population that's within the shown gate. **(A)** Full stain and isotype control for CD69 within EM CD4^+^ T cell (left panels) and EM CD8^+^ T cell (right panels) populations.**(B)** Full stain and isotype controls for CD38 and HLA-DR within EM CD4^+^ T cells (left panels). **(C)** Full stain and isotype controls for CD38 and HLA-DR within EM CD8^+^ T cells (left panels). **(D)** Full stain and isotype control for PD-1 within EM CD4^+^ T cell (left panels) and EM CD8^+^ T cell (right panels) populations.
(EPS)

S4 Fig Gating strategy for flow cytometric intracellular cytokine staining. Shown are representative dot plots for patient 2. **(A)** Samples were analyzed using the following gating strategy for identifying CD8^+^ and CD4^+^ T cells responses: Live gate (SSC versus NEAR IR viability stain) → Singlet gate (FSC-A vs. FSC-H) → Lymphocyte gate (SSC-A vs. FSC-A) → CD8^+^ or CD4^+^ T cells (CD8^+^ vs. CD4^+^) and then CCR7 versus CD45RA to define memory subsets within both the CD8^+^ and CD4^+^ T cell populations; naïve (CD45RA^+^CCR7^+^), central memory (CM) (CD45RA^-^CCR7^+^), effector memory (EM) (CD45RA^-^CCR7^-^) and terminally differentiated (TD) (CD45RA^+^CCR7^-^). Finally, within each of the CD8^+^ and CD4^+^ memory subsets we gated on CD8^+^ and CD4^+^ respectively versus IFNγ, TNFα and IL2. **(B)** Response to stimulation with HIV-gag-peptides; **(C)** Un-stimulated and **(D)** staphylococcal enterotoxin b (SEB) for EM CD8^+^ T cells and EM CD4^+^ T cells. Numbers represent percentage of the shown population that's within the shown gate.
(EPS)

S5 Fig The effect of romidepsin treatment on Staphylococcal enterotoxin B-responsive CD8^+^ and CD4^+^ T cells. Flow cytometric characterization of HIV-gag-specific CD8^+^ and CD4^+^ T cells within the memory subsets at baseline (Base, *n* = 6), on treatment (ROMI, *n* = 5) and at follow-up (Post, *n* = 6). **(A)** Percentages of EM and TD; **(B)** CD8^+^ T cells producing only IFNγ or both IFNγ and TNFα. **(C, D)** Median fluorescence intensity (MFI) for IFNγ and TNFα for SEB-responsive EM **(E)** CD8^+^ T cells and TD **(F)** CD8^+^ T cells. **(G)** Percentages of triple cytokine producing memory EM CD4^+^ T cells producing IFNγ, TNFα and IL-2. **(H, I, J)** MFI for IL-2, TNFα and IFNγ for triple cytokine producing SEB-responsive EM CD4^+^ T cells shown in **(G)**. TD, terminally differentiated; EM, effector memory. Horizontal bars show median values. Statistical comparisons were performed using Wilcoxon matched-pairs signed-ranks test, Asterisk indicate p<0.05.
(EPS)

S6 Fig HIV-1 T cell immunity before and after romidepsin treatment as determined by ELIspot. PBMCs stimulated in triplicate wells with 15-mer peptide pools of15-mer pool for p24 Gag peptides. Mean SFU per 10^6^ shown for baseline and day 84 (70 days after the last romidepsin infusion). One patient who had an invalid ELIspot result on day 84 is not included in the graph. SFU, spot-forming units.
(EPS)

S1 Table Quantitative viral outgrowth assay outcomes. (DOCX)

S1 Checklist TREND Checklist. (PDF)
