# Supplementary figures and images for: The Depsipeptide Romidepsin Reverses HIV-1 Latency In Vivo
Source: PLoS Pathog. 2015 Sep 17;11(9):e1005142. doi: 10.1371/journal.ppat.1005142 (PMC4575032; doi:10.1371/journal.ppat.1005142)

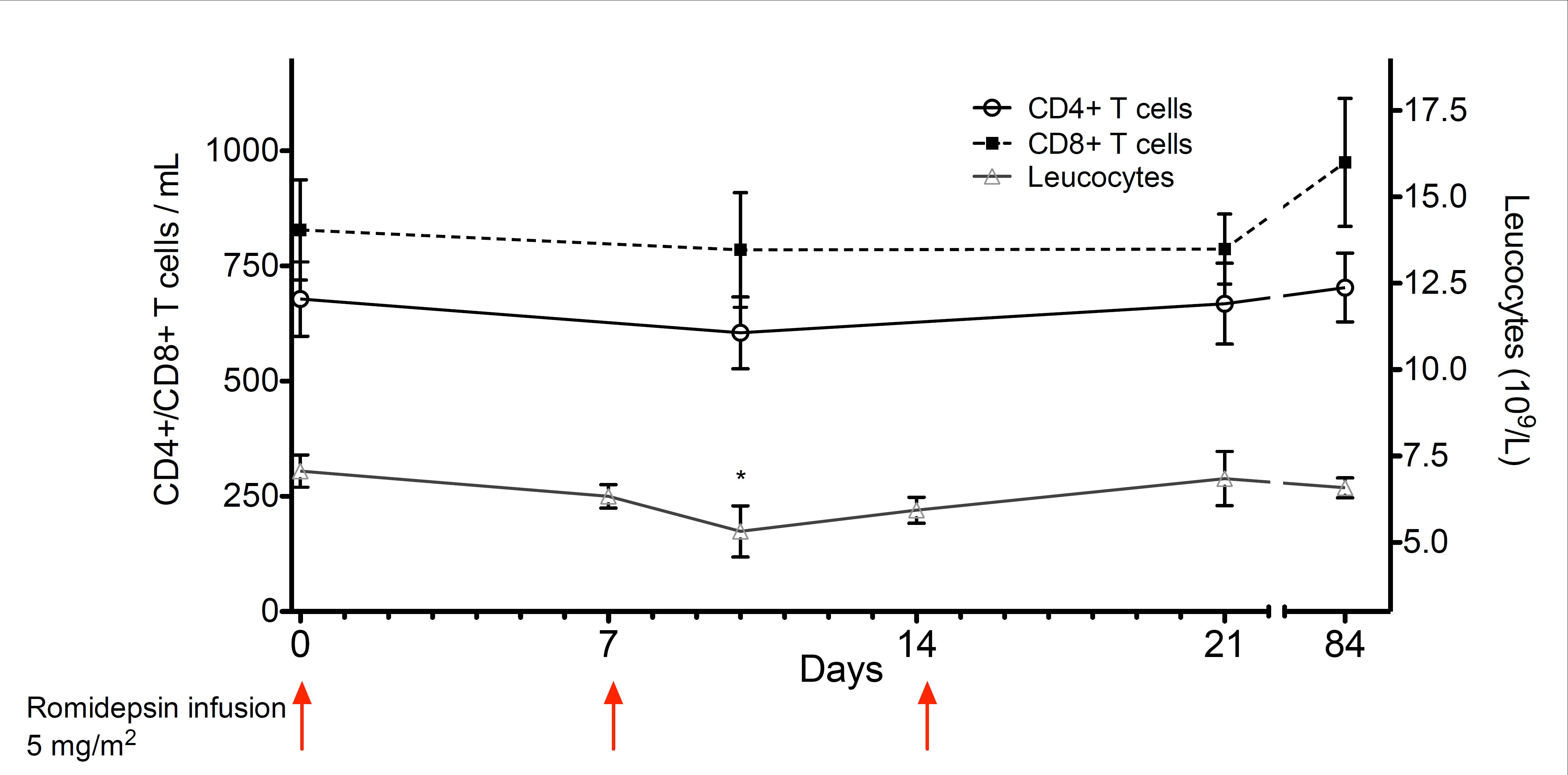

Supplement: S2 Fig — Wilcoxon matched-pairs signed-ranks test, Asterisk indicate p<0.05. (TIF) [file ppat.1005142.s002.tif]
