# Supplementary material for: The Depsipeptide Romidepsin Reverses HIV-1 Latency In Vivo
Source: PLoS Pathog. 2015 Sep 17;11(9):e1005142. doi: 10.1371/journal.ppat.1005142 (PMC4575032; doi:10.1371/journal.ppat.1005142)
Supplement: S1 Table — (DOCX) [file ppat.1005142.s007.docx]

**S1 Table. Quantitative Viral Outgrowth Assay Outcomes.**

|  |  | | |  |  |  |  |  |
| --- | --- | --- | --- | --- | --- | --- | --- | --- |
|  | Baseline | | | After Romidepsin | | | | |
| Patient ID | # of wells | # of HIV+ wells | IUPM (95% CI) | # of wells | | # of HIV+ wells | IUPM (95% CI) |  |
| 1 | 840 | 1 | **0.06** (0.01 to 0.42) | 616 | | 2 | **0.16** (0.04 to 0.65) |  |
| 2 | 312 | 4 | **0.65** (0.24 to 1.72) | 176 | | 1 | **0.28** (0.04 to 2.02) |  |
| 3 | 384 | 0 | **<0.13** (0.2 to 0.93)* | 472 | | 0 | **<0.11** (0.01 to 0.75)* |  |
| 5 | 312 | 2 | **0.32** (0.08 to 1.29) | 352 | | 6 | **0.86** (0.39 to 1.91) |  |
| 6 | 296 | 3 | **0.51** (0.16 to 1.58) | 126 | | 1 | **0.06** (0.40 to 2.83) |  |
| 7 | 136 | 0 | **<0.37** (0.05 to 2.62)* | 312 | | 0 | **<0.16** (0.02 to 1.14)* |  |
| * Assay limit of detection with 95% CI is the value calculated if a single well had been HIV positive. | | | | | | | | |
